# Supplementary material for: High-Throughput Analysis of Arabidopsis Stem Vibrations to Identify Mutants With Altered Mechanical Properties
Source: Front Plant Sci. 2018 Jun 12;9:780. doi: 10.3389/fpls.2018.00780 (PMC6005829; doi:10.3389/fpls.2018.00780)
Supplement: Supplementary file 9 [file Presentation_1.PDF]

Supplementary Material

High-throughput Analysis of Arabidopsis Stem-free Vibrations to Identify Mutants with Altered Mechanical Properties

Miyuki T. Nakata, Masahiro Takahara, Shingo Sakamoto, Kouki Yoshida, Nobutaka Mitsuda*

*** Correspondence:** Nobutaka Mitsuda: nobutaka.mitsuda@aist.go.jp

1 Supplementary Data

Development environment for AraVib and AraVibS

Here we show an example of environment construction in the case of MacOS (MacOS Sierra with Xcode 8.3.3). Even when using Linux and Windows, this script can be executed by installing *ffmpeg*, Python-distribution Anaconda and OpenCV-Python. The packages/libraries and their versions are ffmpeg (3.3.4), homebrew (1.3.7), pyenv (1.1.3), Python 3 (3.6.1), Conda (4.3.30), Jupyter notebook (5.0.0), JupyterLab (0.27.0), Matplotlib (2.0.2), Numpy (1.13.1), OpenCV-Python (3.3.0.10), Pandas (0.20.3), Scipy (0.19.1), Sqlite (3.13.0) and Scikit-learn (0.19.1). Our script may be applicable to other versions.

Firstly, Homebrew, a useful package manager for MacOS users, was installed according to the instruction of the web page (https://brew.sh/). Homebrew works as a command *brew* in Terminal on MacOS. *pyenv*, a Python version manager, was installed by the following Terminal command.

$ brew install pyenv

You can find how to install *pyenv* in your environment in the GitHub page (https://github.com/pyenv/pyenv). *ffmpeg* was installed by the *brew* command.

$ brew install ffmpeg

For easy installation of Python 3 and its data-science packages, we used the open source Anaconda Distribution (https://www.anaconda.com/). We installed Anaconda by the *pyenv* command in Terminal.

$ pyenv install anaconda3-4.4.0

Conda, Jupyter notebook, Matplotlib, Numpy, Pandas, Scipy, Sqlite and Scikit-learn are packaged in Anaconda.

To put the Python package installed by the *pip* command directly under Anaconda, the next command was executed.

$ conda install -c anaconda pip

After that we installed JupyterLab (https://github.com/jupyterlab/jupyterlab) and OpenCV-Python (https://docs.opencv.org/3.0-beta/index.html) with the *pip* command.

$ pip install jupyterlab

$ pip install opencv-python

Development of AraVib

This section shows the details of AraVib. This script is divided into four parts:

1. Obtaining a set of images from a movie file by dividing into frames

2. Detection of the marker of the stem apex by thresholding from a set of images

3. Identification of centroid coordinates of the marker and calculation of displacement

4. Determination of the stem vibration frequency by the FFT analysis

The schematic views are shown in Supplementary Figure 1 to 3.

For division of the 3-second movies captured with the iPhone/iPad image sensor into 720 single-frame image files by *ffmpeg*, in the Terminal window enter the following command:

$ ffmpeg -i <*the path of .mov file*> -ss 00:00:00 -t 00:00:03.00 -vcodec png image_%03d.png

For automation of conversion from movie file to image file, we executed the *ffmpeg* command by *shlex* and *subprocess* modules of Python 3 and the list of the movie files was handled with the functions of Pandas and Sqlite.

To detect the red paint at the apical part of the stem, *cv2* module (OpenCV-Python) was imported. Firstly, a set of the single-frame images was read by *cv2.imread* function and converted into the HSV images (**H**ue, **S**aturation and **V**alue) by *cv2.cvtColor* function. In order to remove noise, the HSV images were blurred by *cv2.blur* function, and binarized based on the thresholds set for each of H, S and V channels. Appropriate values of the threshold of S and V were determined by experiments in which actual 46 movie files were analyzed (see below for details). H, S and/or V threshold-based binarized images were combined by logical AND. In order to further remove noise and mitigate the influence of gap due to ambiguity, the combined binary images were eroded/dilated by Morphological operators *cv2.erode* function and *cv2.dilate* function, respectively. Finally, the final binary images were saved with *cv2.imwrite* function.

To obtain the centroid coordinates of the red paint, the values of the image moments of the integrated binary images were calculated by *cv2.findContours* and *cv2.moments* function. Details are described in the web page of OpenCV-Python tutorials about how to obtain the centroid coordinates from the values of the image moments (http://opencv-python-tutroals.readthedocs.io/en/latest/py_tutorials/py_imgproc/py_contours/py_contour_features/py_contour_features.html). The processing method for each detected number of objects is as follows:

- In the case of 1, the centroid coordinate of the single object was accepted unconditionally.

- In the case of 2 or more, the centroid coordinates closest to that of the previous time point was accepted. To calculate the distance between coordinates, *numpy.linalg.norm* function was used (Supplementary Figure 5B).

- In the case of 0, NA was temporarily assigned to the centroid coordinates. After the centroid coordinates of all images were calculated, NA data was removed by substituting the average values before and after it by using *numpy.roll* function and *numpy.where* function. In the case where the images without detected object are continuous, the centroid coordinates of the detected objects at the two closest time points were equally divided. Details are show in the next section.

To evaluate the validity of the detected centroid coordinates, the original image was overwritten with a circle with the *cv2.circle* function.

To calculate the displacement, the centroid coordinates of the starting frame were taken as the origin and relative coordinates were determined. The displacement was calculated from the relative coordinates with NumPy matrix operation function and *numpy.linalg.norm* function. Individual displacement was normalized with the median of all displacements. To determine the major frequency of the stem, a vibration data was resolved into its frequency components by the Fast Fourier Transform (FFT). Frequency analysis of vibration data was performed using the function of FFT implemented in SciPy (Supplementary Figure 6A). However, noises in raw vibration data made the power in low frequency band extremely high ("FFT(raw)" in Supplementary Figure 6B). Thus, normalized displacement was processed for proper frequency analysis before the FFT analysis. As a result of examination by simulation (details are described in the next section), it was found that application of Hanning window lead to good results. Hanning window was applied to vibration of 1 second from the starting point of the vibration. The starting point was the time point at which the difference between the displacement of two sequential frames was the largest. Application of the Hanning window was done with the *numpy.hanning* function.

To calculate the frequency from the displacement, *fftpack.fftfreq* and *fftpack.fft* functions of SciPy were used. Because frequencies below 2 Hz tended to exhibit nonspecifically high values, the frequency of the highest power among the frequencies greater than 2 Hz was defined as the vibration frequency of the stem. Data of the calculated frequencies were saved in the database with the function of Sqlite immediately. Lastly, the frequency data saved in the database were output as a csv file by the function of Pandas. This Python script is attached as "vibration_freq_v9.py".

Parameter and algorithm optimization

For robust frequency estimation, we examined the following three topics; optimization of threshold values for detection, completion of missing values and preprocessing displacement data before FFT analysis.

For optimization of threshold values for detection, the image from the RGB color space was converted into the HSV color space and divided it into H, S and V. Because less than 5 and more than 165 in H (shown as H = [5, 165] in Supplementary Figure 1B and later) corresponds to red, the images were filtered with these values as thresholds (Supplementary Figure 1B). Next, threshold values of S and V were optimized to detect only marker in most images (Supplementary Figure 1B). To this end, we used the vibration images for 46 individuals of a wild type Col-0 obtained by three independent experiments (Supplementary Figure 1C). By changing parameters progressively, the ratio of successful detection was achieved close to 100% when the parameters were set to H = [5, 165], S = 60 and V = 90. Supplementary Figure 1D shows two examples of binarization. Supplementary Movie 3 shows an example of the successfully processed movie.

For completion of missing values, object detection from the processed image and calculation of the centroid coordinates and displacement were performed by OpenCV-Python and NumPy (Supplementary Figure 2A-B). Supplementary Movie 4 shows an example of the slow-motion video with detected centroid coordinates as the center of red circles. The distance from the first frame image was calculated to be the displacement. For the frames with no object detection (NOD) (Supplementary Figure 2C), approximated values were interpolated by NumPy *roll* function to enable Fast Fourier Transform (FFT) analysis, which will be described later. The NOD frames occupy 20% of movies of Col-0, 10% of *nst1-1 nst3-1* and 35% of *wrky12-1* (Supplementary Figure 2C) and were sometimes found in the first part of the movie when the moving speed of the marker was fastest (Supplementary Figure 2D). To verify whether complementation of missing value is appropriate, we conducted the following three verifications in cases of two and four consecutive missing values; manually plotting missing values on the vibration graph (the dotted line of magenta in Supplementary Figure 2D), estimating the position of the centroid coordinates on the image (Supplementary Figure 2E), and a simulation (Supplementary Figure 2F, G). In the simulation, we replaced the several centroid coordinates with the missing value. The estimated centroid coordinates were compared with the true values (Supplementary Figure 2F). The true value of the displacement and the estimate showed high correlation (Supplementary Figure 2G). From these results, we concluded that our approximation method is appropriate.

For preprocessing displacement data before FFT analysis, two different pretreatments were examined (Supplementary Figure 3A-B). In the first method, all displacement values before the start were replaced with 0 in order to eliminate the influence of temporal change of the marker before vibration. The second method is to apply the Hanning window to the vibration data of 1 second from the start point. Hanning window is a window function used for harmonic analysis as described in the main text. In order to validate them, a simulation using artificial model data of damped free vibration was carried out. The equation for generating the ideal artificial model data of damped free vibration model data (y_ideal) is as follows:

$$y\_ideal = e^{-0.2(x-10)} \cos 4(x-10) \quad \text{(Eq. S1)}$$

The equation for generating artificial model data including Gaussian error (y) is as follows:

$$y = e^{-0.2(x-10)} \cos 4(x-10) + Gaussian\_error \quad \text{(Eq. S2)}$$

In the ideal model data, the displacement before the start time was set to 0, and in the model data including the Gaussian error as a noise, in order to reproduce the actual situation, the displacement change before the start time was altered so that it resembles the actual data. These model data were repeatedly generated and analyzed by the function of NumPy. The output generated by FFT of the model data (with error) actually showed a power spectrum analogous to that obtained by FFT of raw data (Supplementary Figure 3C). And then, repeatedly generated 10,000 model data (with error) were processed by the above-mentioned two methods (Supplementary Figure 3C) and the major frequency was determined by FFT analysis. As a result, the false estimation rates of the first and second methods were 1.06 % and 0.01%, respectively. Supplementary Figure 3C graphically shows overlay of the 1,000 results. In the case of the first method, the variation of the frequency peak was broad, whereas less variation in the second method and the shape of the frequency peak was similar to that of the ideal data (Supplementary Figure 3C). Application of the Hanning window to the actual data enabled to detect the main frequency as a sharp peak (Supplementary Figure 3B).

Supplementary Movie 1. The high speed-shooting video (240 fps) of the damped free vibration of a Col-0 inflorescence stem.

Supplementary Movie 2. Slow-motion video (30-fps) of Supplementary Movie 1.

Supplementary Movie 3. Slow-motion video (30-fps) of binarized images of Supplementary Movie 1.

Supplementary Movie 4. Supplementary Movie 2 with a red circle indicating the detected centroid coordinate.

Supplementary Movie 5. The high speed-shooting video (240 fps) of the damped free vibration of a *nst1 nst3* inflorescence stem.

Supplementary Movie 6. Slow-motion video (30-fps) of Supplementary Movie 5.

Supplementary Movie 7. The high speed-shooting video (240 fps) of the damped free vibration of a *wrky12* inflorescence stem.

Supplementary Movie 8. Slow-motion video (30-fps) of Supplementary Movie 7.

2 Supplementary Figures and Tables

2.1 Supplementary Figures

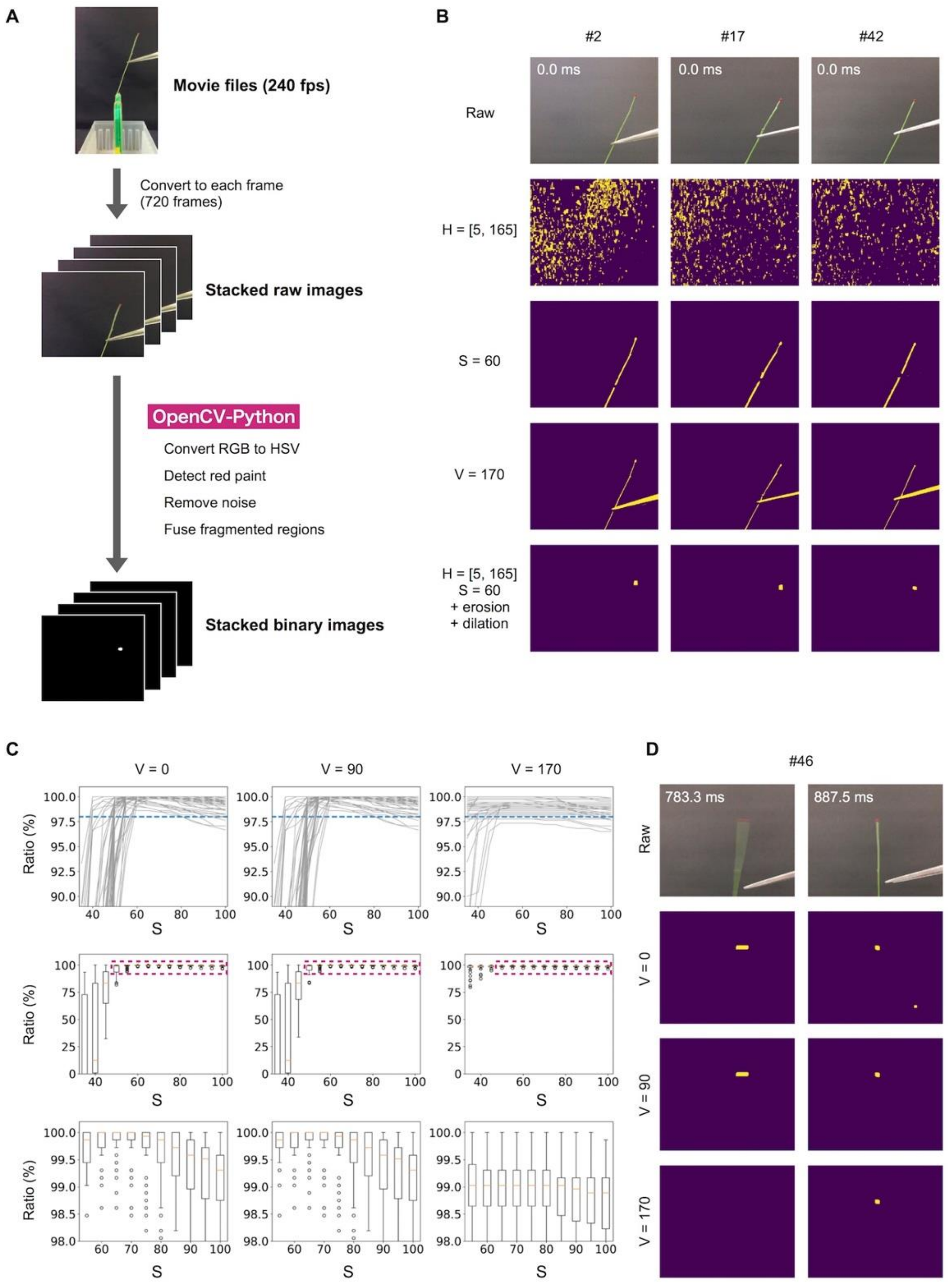

Supplementary Figure 1. Binarization of images and parameter optimization for red marker detection. (A) Schematic diagram of division of movie files and binarization of images. (B) Binary images based on the values of H, S and V in three independent experiments and the result by H-S threshold and morphological operation. H = [5, 165] means that if H < 5 or H > 165 then the pixels

are 1 (show in yellow), else 0 (shown in black). S = 90 means if S > 90 then the pixels are 1, else 0. V = 170 means that if V > 170 then the pixels are 1, else 0. +erosion+dilation means that morphological operation (erosion and dilation) was applied to the binary images. (C) Percentage of a single object detection when nine different S values and three different V values were used as threshold values. For 46 wild-type samples, the ratio of frames with a single object to total frames was examined. Morphological operation (erosion and dilation) was done before the object detection. The top columns are line graphs obtained by overlaying the results from all samples. The middle columns are boxplots showing the distribution for each S value. The bottom columns are magnified graphs of the dashed region at the middle columns. (D) The example of images calculated by H = [5, 165] S = 90 and morphological operation (erosion and dilation) when three different V values are used as threshold values.

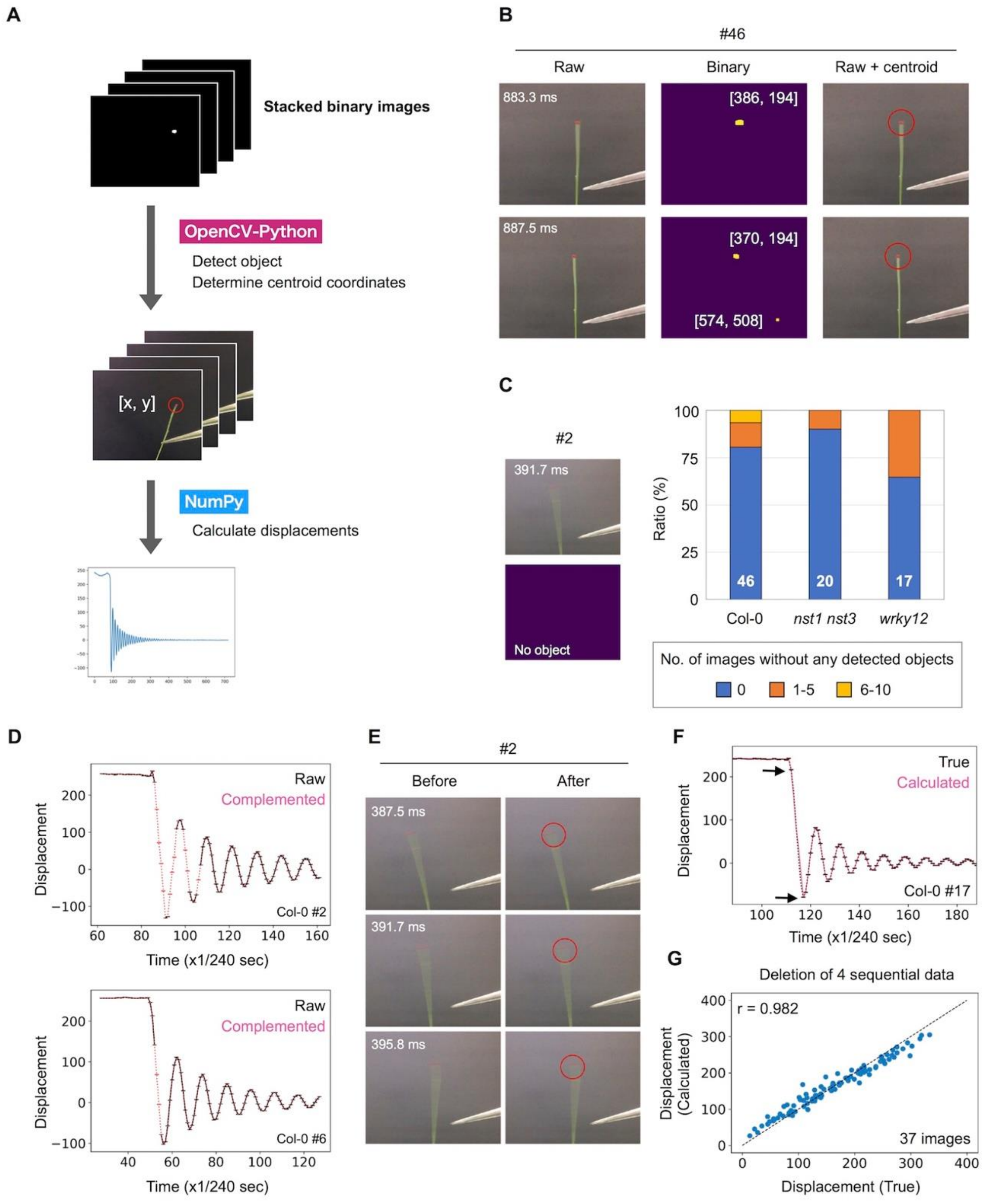

Supplementary Figure 2. Calculation of vibration data of displacement from binarized image. (A) Outline of program for calculation of displacements. (B) An example of images with multiple objects. The centroid coordinates of objects detected are overwritten on the binary image. On the right columns, circles are drawn around the centroid coordinates of the adopted object on the raw image. The bottom binarized image is the same as the image shown in Supplementary figure 4d (V = 0, 887.5 ms). (C) Percentage of samples including images with no object detection (NOD). Of the

720 images, samples including 1-5 NOD images are shown in orange, samples including 6-10 NOD images are shown in yellow, and samples in which one or more objects are detected in all images are shown in blue . The number in the graph is that of samples of each strain. Threshold is H = [5, 165] S = 60 V = 90. (D) Two examples of vibration data of displacements including NOD data. The actual data is shown as black and the complemented waveform is indicated by magenta. (E) Three examples of the NOD images before and after complementation. (F) An example of vibration data of displacements without NOD data. The actual data is shown as a black dashed line. Black short horizontal lines show displacement values after artificial deletion. The complemented waveform is indicated by magenta. The arrows represent points just before and after the sequential artificial defects. (G) A scatter diagram of true values and the calculated values when four consecutive data are deleted from the centroid coordinates. The number of samples without any NOD images used in this analysis is 37. *r* represents the Pearson's correlation coefficient.

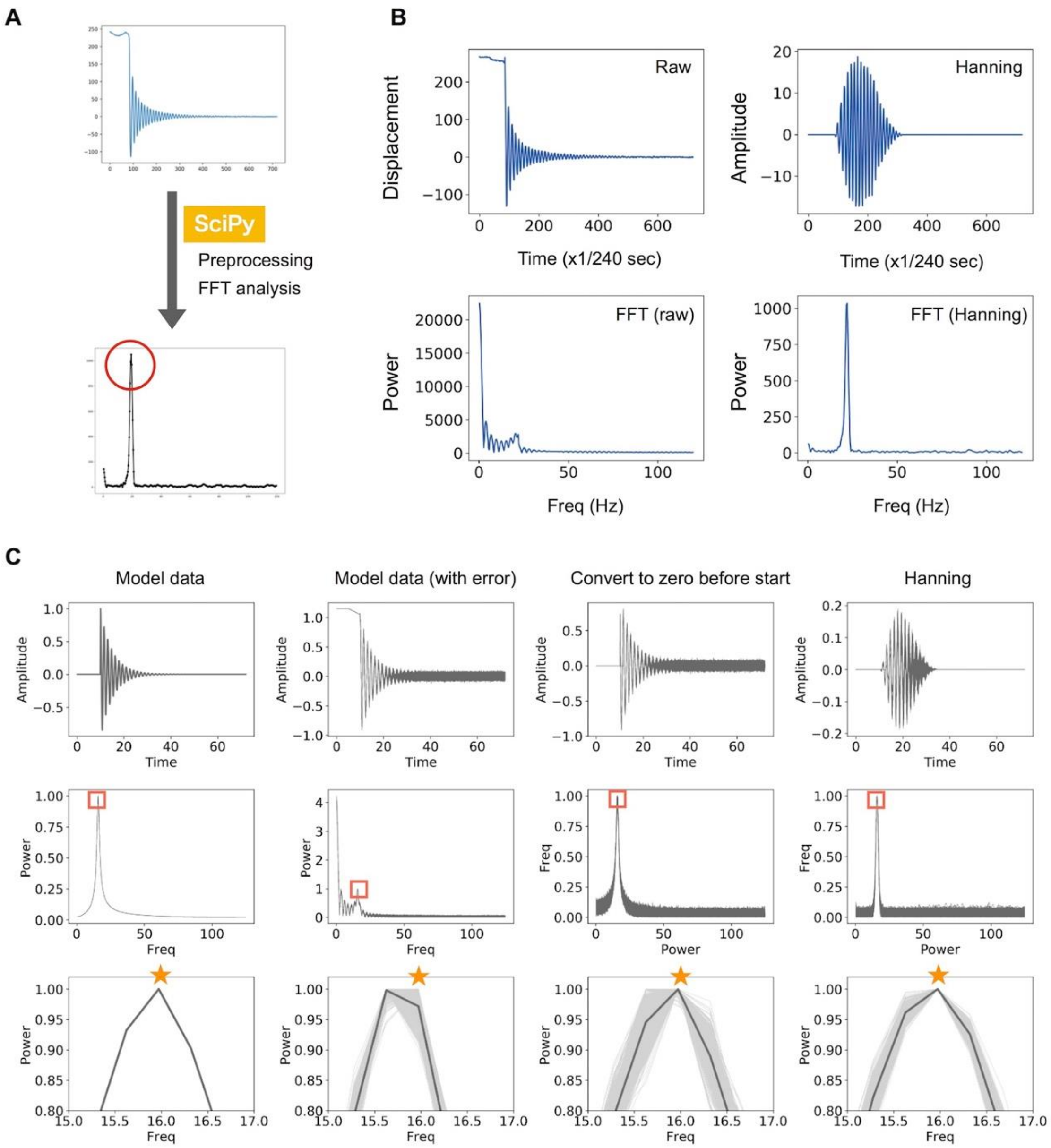

Supplementary Figure 3. Preprocessing of FFT analysis. (A) The procedure of the frequency calculation. (B) Vibration data and FFT power spectrum before and after preprocessing of actual sample. "Raw" means before preprocessing and "Hanning" means after preprocessing by Hanning window. (C) Simulation using model data. The top columns show vibration data, the middle columns show FFT power spectrum and the bottom columns show magnified graphs of the region surrounded with the red square in the middle columns. Stars indicate the frequency showing the maximum power of model data (without error).

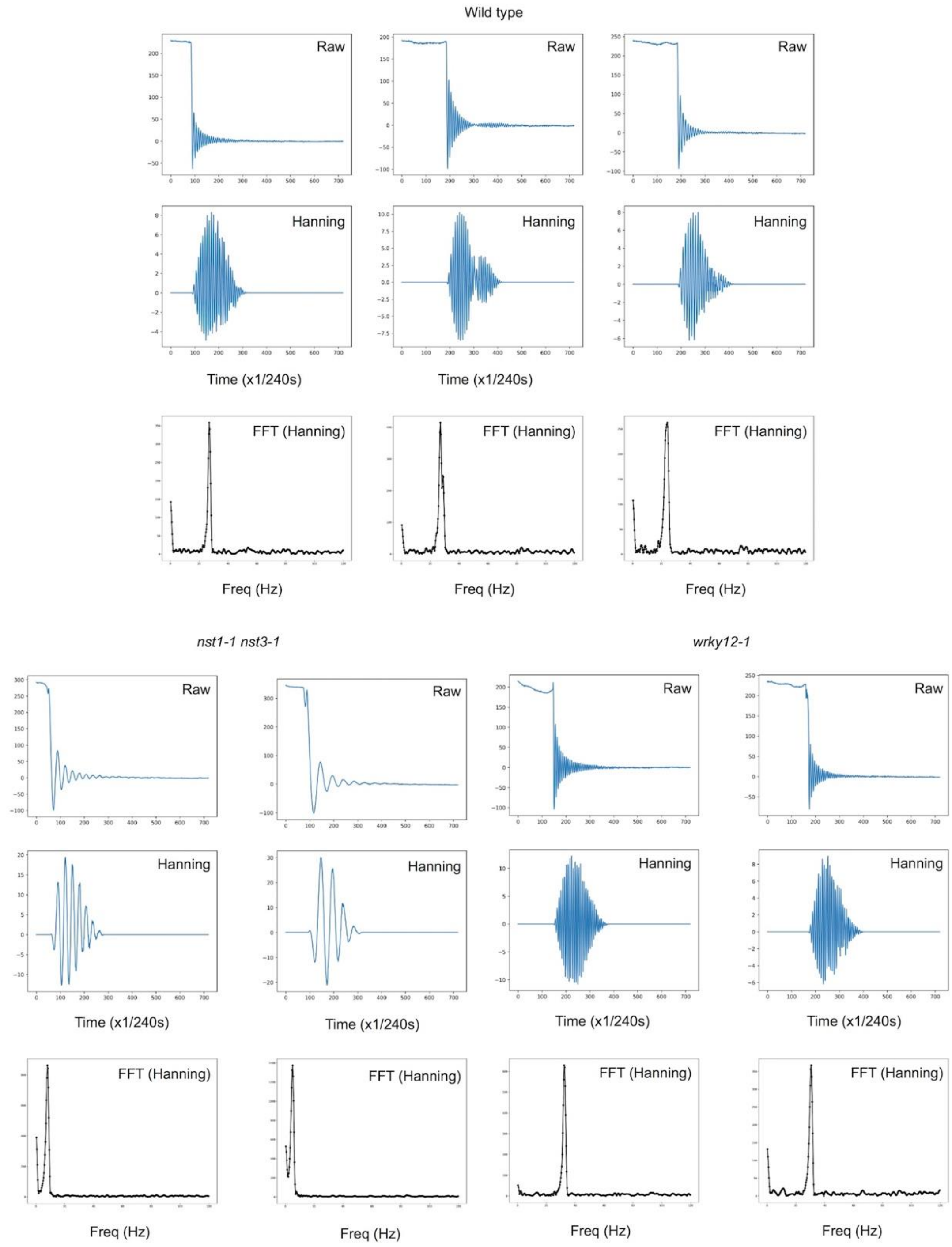

Supplementary Figure 4. Examples of vibration waveform data (Raw), waveform data (Hanning) after Hanning window conversion, and FFT power spectrum (FFT) of each genotype. Data of samples different from those in Figure 2 are shown.

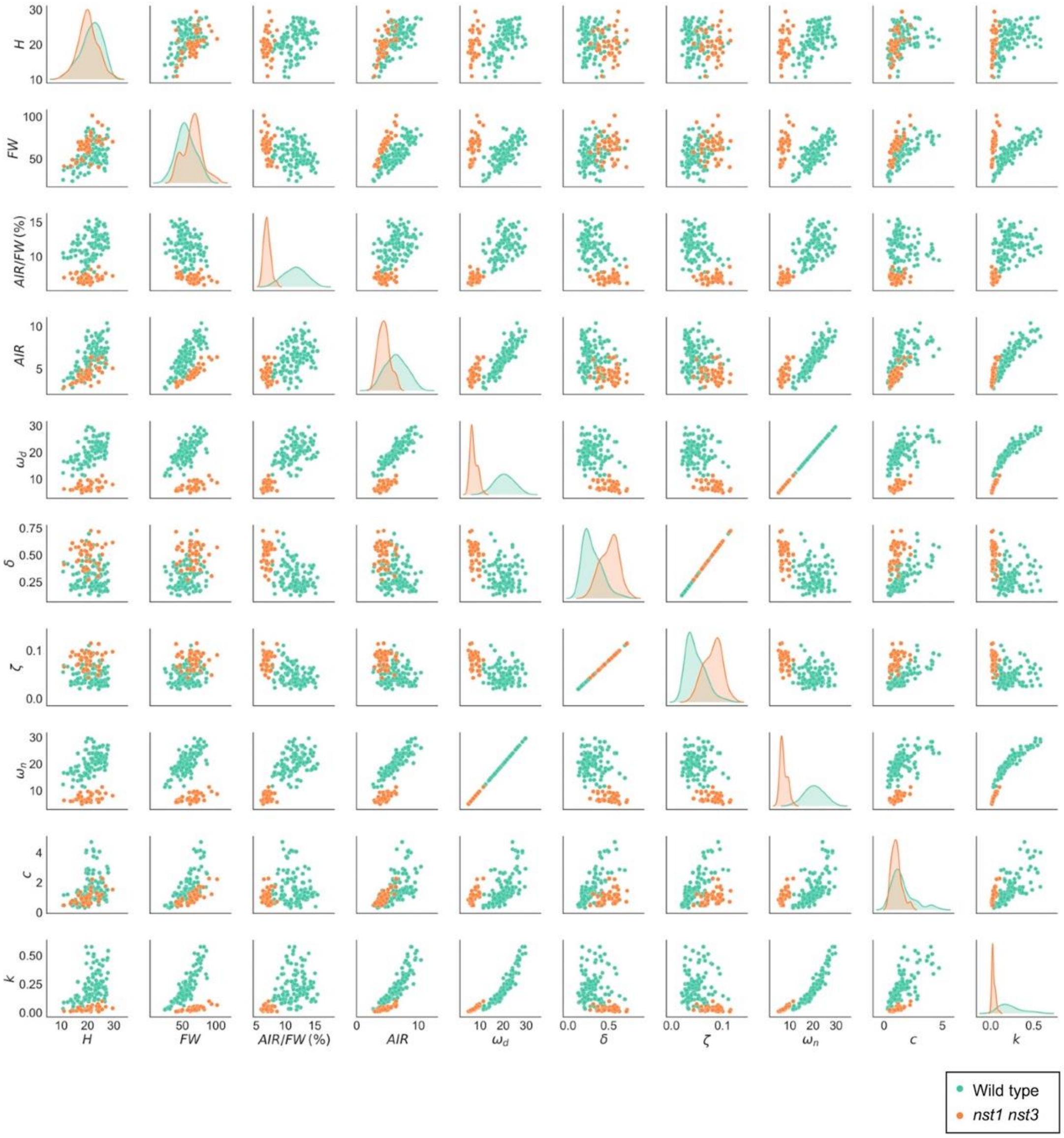

Supplementary Figure 5. Scatter plots of all parameter pairs and density plots of all parameters of wild type and *nst1 nst3* in condition 1. The number of samples are 111 (wild type) and 41 (*nst1 nst3*).

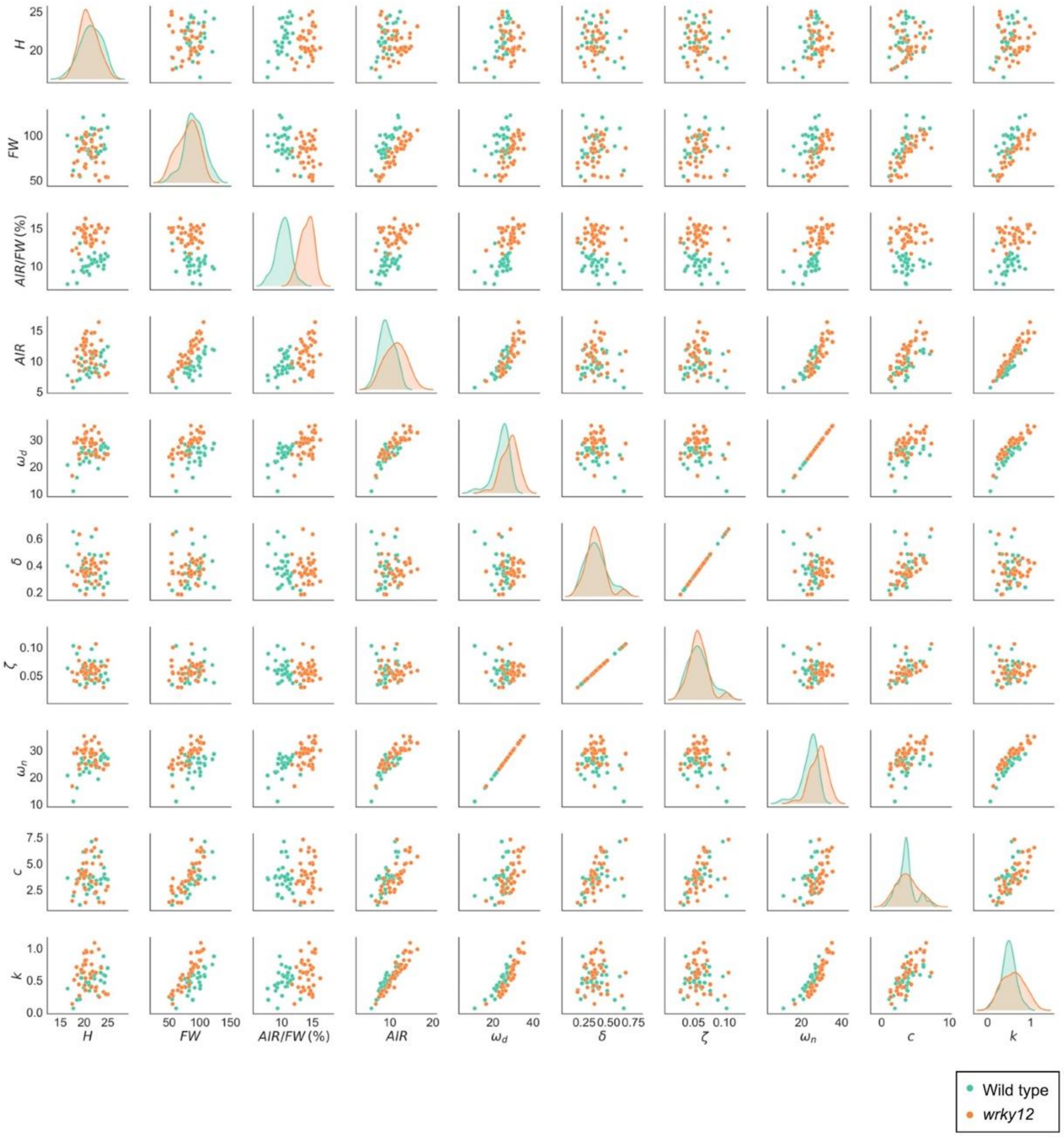

Supplementary Figure 6. Scatter plots of all parameter pairs and density plots of all parameters of wild type and *wrky12* in condition 2. The number of samples are 29 (wild type) and 37 (*wrky12*).

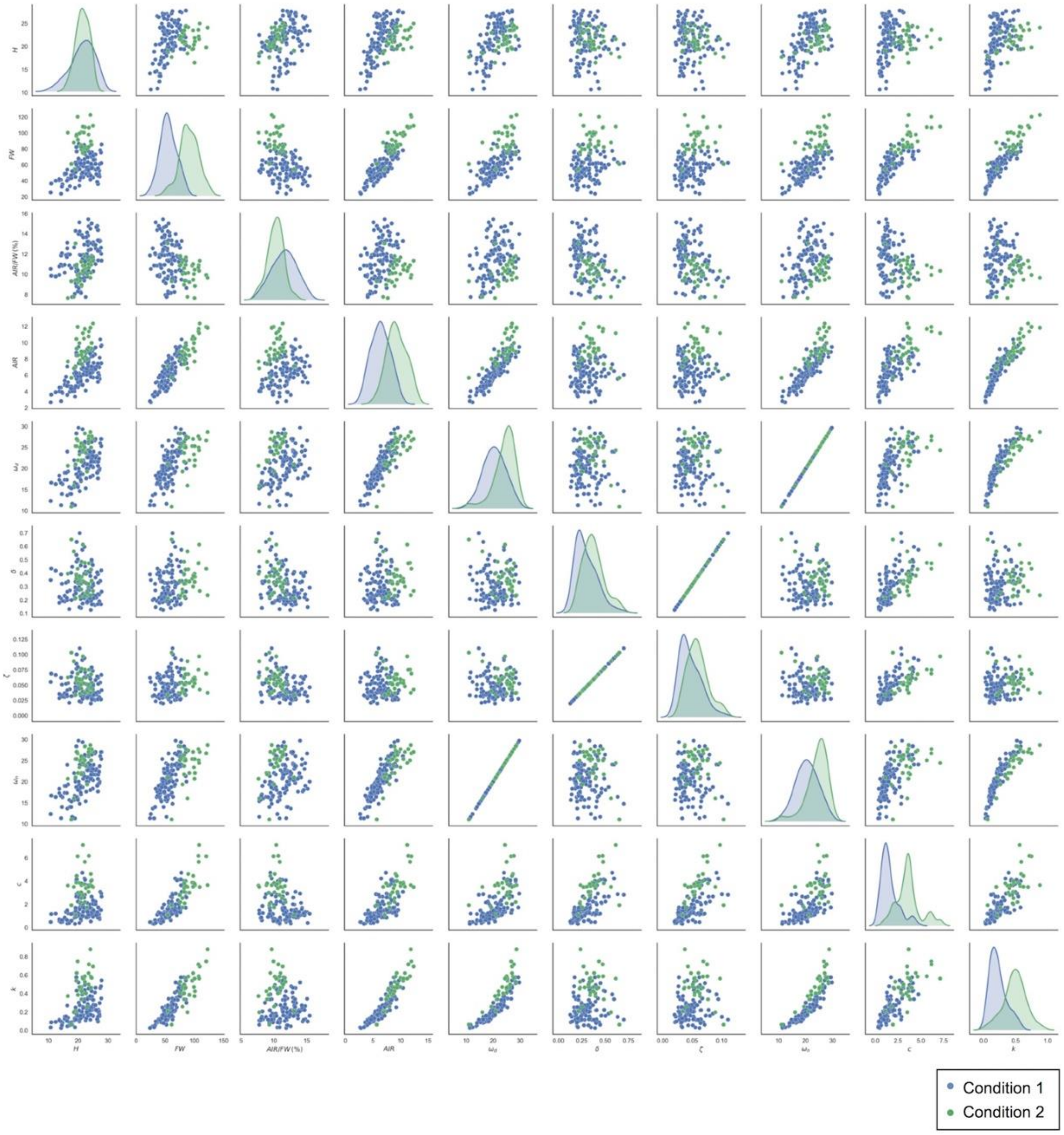

Supplementary Figure 7. Scatter plots of all parameter pairs and density plots of all parameters of wild type in two different conditions. The number of samples are 111 (condition 1) and 29 (condition 2).

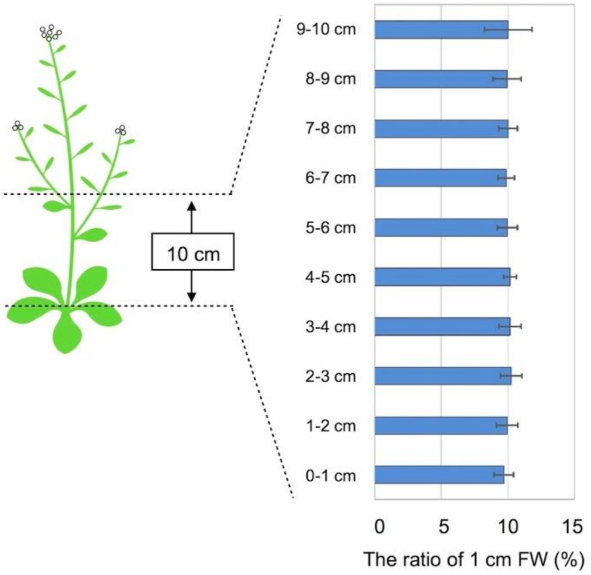

Supplementary Figure 8. Preparation for vibration test. Measured value of weight distribution in longitudinal direction of stem. Data represent the mean ± standard deviation. The number of samples is 17.

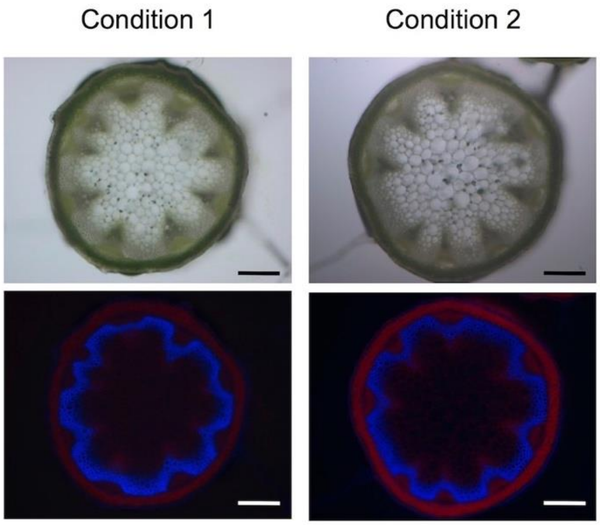

Supplementary Figure 9. Differences between wild-type stems grown in condition 1 and condition 2. Hand cross sections of 1.5-cm basal position of the inflorescence stems. Bright-field (top) and UV autofluorecence images (bottom) are shown. Scale bars, 200 µm.

Supplementary Table 1 Approximation of logarithmic decrement in damping vibration curve of inflorescence

Line	Growth condition	Slope	Correlation coefficient (r^2)	Regression analysis with c-test (*p*-value)	n
Col-0	1	-0.300±0.011[#1]	0.946-1.000[#2]	0.0000-0.0274	111
nst1 nst3		-0.509±0.017[*]	0.962-1.000	0.0001-0.0191	41
Col-0	2	-0.374±0.021	0.930-0.999	0.0005-0.0358	29
wrky12		-0.369±0.017	0.955-1.000	0.0002-0.0230	37

#1:Mean±Standard Error, #2:Minimum-Maximum, *:Significant at p<0.001 (t-test)

Supplementary Table 2 Growth-related traits in the harvested inflorescence-stems of Arabidopsis

Line	Growth condition[#1]	Stem height (cm)	Status of 10cm-length stem explants: Fresh Weight (FW, mg)	Status of 10cm-length stem explants: Alcohol-Insoluble Residue (AIR, mg)	Status of 10cm-length stem explants: Ratio AIR / FW (%)	Equivalent mass of modeled beam ($m_{eq,}$ mg)	Initial angle (°)	n
Col-0	1	21.5±0.4[#2]	55.6±1.3	6.4±0.2	11.6±0.2	12.3±0.3	20±3[#3]	111
nst1 nst3		20.2±0.6	65.2±2.2***	4.5±0.1***	6.9±0.1***	14.4±0.3***	22±4	41
Col-0	2	21.5±0.4	91.3±2.9	9.3±0.3	10.3±0.2	20.1±0.6	21±3	29
wrky12		21.0±0.3	80.3±2.9**	11.3±0.4***	14.1±0.2***	17.7±0.7**	23±3	37

#1:See Material and Methods, #2:Mean±Standard Error, #3:Mean±Standard Deviation, *:Significant at $p<0.05$, **:Significant at $p<0.01$, ***:Significant at $p<0.001$ (Welch's t-test)
